# No Association between Elevated 2-h Postprandial Blood Glucose Levels and Functional Outcomes of Small-Artery Occlusion in Patients with Diabetes

**DOI:** 10.3389/fneur.2018.00093

**Published:** 2018-02-26

**Authors:** Ji Liu, Dongzhe Hou, Yuan Gao, Jialing Wu

**Affiliations:** ^1^Department of Medicine, Tianjin Huanhu Hospital, Tianjin Key Laboratory of Cerebrovascular and Neurodegenerative Diseases, Tianjin, China; ^2^Department of Neurology, Tianjin Huanhu Hospital, Tianjin Key Laboratory of Cerebrovascular and Neurodegenerative Diseases, Tianjin, China; ^3^Department of Neurorehabilitation, Tianjin Huanhu Hospital, Tianjin Key Laboratory of Cerebrovascular and Neurodegenerative Diseases, Tianjin, China

**Keywords:** 2-h postprandial blood glucose, diabetes, small-artery occlusion, outcome, stroke

## Abstract

**Background:**

The association between 2-h postprandial blood glucose level (2hPBG) and functional outcomes in patients with small-artery occlusion (SAO) is poorly understood. We aimed to explore the relationship between 2hPBG levels and functional outcomes in SAO patients with diabetes.

**Methods:**

We retrospectively analyzed 174 diabetic patients diagnosed with SAO, and 2hPBG values were classified into four groups according to quartiles (<8.90, 8.90 to <12.16, 12.16 to <15.14, and ≥15.14 mmol/L), or according to clinical glycemic recommendations for adults with diabetes (<10 and ≥10 mmol/L, respectively). The relationship between 2hPBG levels and modified Rankin Scale (mRS) scores was assessed using univariate and multivariate analyses.

**Results:**

Among all patients with SAO, there were 139 patients with favorable outcomes and 35 patients with poor outcomes. National Institutes of Health Stroke Scale scores were significantly different according to mRS scores (*P* < 0.001) in both the univariate and multivariate analyses. The binary logistic regression analyses showed that compared with the lowest quartile (<8.90 mmol/L), elevated 2hPBG levels (8.90 to <12.16, 12.16 to <15.14, and ≥15.14 mmol/L) were not associated with mRS scores after adjusting for multiple confounding factors. Compared with patients with 2hPBG levels <10 mmol/L, those with 2hPBG levels ≥10 mmol/L did not have a significant risk of poor outcome after adjusting for confounders. Meanwhile, the negative results appeared in the ordinal logistic regression of 2hPBG levels and 3-month functional outcomes.

**Conclusion:**

Elevated 2hPBG levels were not associated with unfavorable functional outcomes 3 months after stroke onset in SAO patients with diabetes.

## Introduction

Small-artery occlusion (SAO) is a subtype of ischemic stroke based on the classification of the Trial of ORG10172 in Acute Stroke Treatment (TOAST), and accounts for 20–30% of ischemic stroke cases in China (1–3). Assessment of functional independence is the most accurate way to evaluate the outcomes of SAO patients because SAO has the lowest stroke morbidity and mortality rate among stroke subtypes (3).

There is increasing evidence that hyperglycemia immediately after stroke and pre-stroke glucose metabolism play key roles in the outcome of stroke patients (4–10). It has been shown that stroke can lead to hyperglycemia in the acute phase, and hyperglycemia at admission is associated with poor outcomes in non-SAO patients (8, 11); however, the association between hyperglycemia and outcome in SAO patients remains controversial (11–13).

In addition to fasting blood glucose (FBG), 2-h postprandial blood glucose (2hPBG) is another indicator used to evaluate glucose metabolism. In China, isolated raised 2hPBG levels have been noted in 85.8% of patients with impaired glucose tolerance after ischemic stroke (14). Some studies showed that atherosclerosis is more strongly associated with 2hPBG levels than fasting glucose levels (15). However, the association between 2hPBG level and functional outcome following SAO, especially among patients with diabetes, has not been established.

Therefore, the aim of the present study was to investigate the role of 2hPBG levels in functional outcomes of SAO patients with diabetes 3 months after stroke onset.

## Materials and Methods

### Patient Selection

This study was approved by the ethics committee of Tianjin Huanhu Hospital. Written informed consent was obtained from the individuals and their families at the beginning of the study. We retrospectively reviewed data from the stroke registry of the Department of Neurology of Tianjin Huanhu Hospital between January 1, 2008, and October 31, 2012.

Diagnosis of acute cerebral infarction was made according to World Health Organization criteria (13). Patients were required to be admitted within 3 days of their first ischemic stroke, and diagnoses were confirmed by brain computed tomography or magnetic resonance imaging. Patients diagnosed with SAO, which was defined according to the TOAST classification criteria (1, 3). The location of infarction was limited in the subcortex or brain stem, with a diameter <1.5 cm as shown on CT or MRI. Exclusion criteria for this study were as follows: (1) age ≤18 years, (2) potential cardiac sources for embolism, (3) stenosis greater than 50% in an ipsilateral artery, (4) unwillingness to participate, and (5) the patients who received r-tPA therapy.

**Figure 1:**
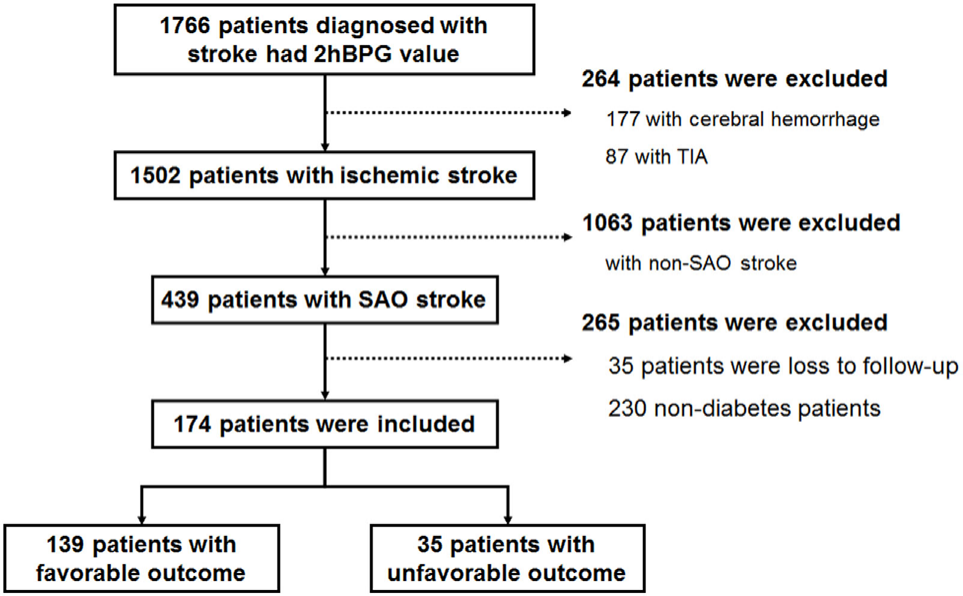
Flow chart of patient selection.

Of 1,766 stroke patients with available 2hPBG data, 1,502 were diagnosed with ischemic stroke, and 439 were diagnosed with SAO. After excluding patients who were lost in follow-up and patients without diabetes, 174 patients with diabetes were analyzed in this study (Figure 1). We classified 2hPBG values into four groups according to quartiles (<8.90, 8.90 to <12.16, 12.16 to <15.14, and ≥12.30 mmol/L). The 2hPBG values also were classified into two groups according to the clinical glycemic recommendations for non-pregnant adults with diabetes (<10 and ≥10 mmol/L, respectively) (16).

### Demographic and Clinical Assessment

Patients’ demographic information and stroke risk factors were obtained from stroke registry records. The National Institutes of Health Stroke Scale (NIHSS) score at admission was used to assess stroke severity (17).

Hypertension was defined as diastolic blood pressure ≥90 mmHg and/or systolic blood pressure ≥140 mmHg; patients currently receiving antihypertensive treatment also were defined as having hypertension (18). Diabetes was defined as an FBG level ≥7.0 mmol/L and/or 2hPBG level ≥11.1 mmol/L, a previous diagnosis of diabetes and/or the use of hypoglycemic agents, and/or an HbA1c level on admission ≥6.5% (10, 19).

Dyslipidemia was defined as a triglyceride level >2.26 mmol/L and/or a total cholesterol level >6.21 mmol/L; patients receiving treatment with cholesterol-reducing agents also were defined as having dyslipidemia (10, 18). The current drinkers were defined that patients who consumed alcohol at least once a week for >1 year. Patients who smoked tobacco products almost every day for >1 year were defined as current smokers. Obesity was defined as a body mass index ≥30 kg/m^2^ (2, 10).

### Laboratory Methods

Fasting blood samples were collected from the cubital vein from each patient in the early morning. The serum lipids were measured on Day 2 after admission. A standard oral glucose tolerance test was performed in all patients on Day 3 or 4 after admission; after overnight fasting, patients drank 250 mL of a solution including 75 g of glucose within 3 min. Immediately before administering the drink and again after 120 min, venous blood samples were collected in sodium fluoride tubes for plasma glucose measurements (14, 19).

### Follow-up and Outcomes Assessment

Patients were followed up through either telephone or in-person interviews. In the current study, the deadline for follow-up was 3 months after stroke. Functional outcomes were evaluated using modified Rankin Scale (mRS) scores. A favorable outcome was defined as an mRS score of 0–2, and an unfavorable outcome was defined as an mRS score of 3–5 after stroke onset (10).

### Statistical Analysis

Continuous variables are presented as means ± SD. The significance of inter-group differences was assessed using the *t*-test and one-way analysis of variance. Categorical variables are presented as counts and percentages. The chi-square test was used to analyze differences between groups.

Univariate and multivariate analyses were used to analyze functional outcomes. We compared the difference between the favorable outcome group and poor outcome group in the univariate analysis. When analyzing mRS scores, we compared the proportion of mRS defined by favorable/unfavorable outcome and the overall distribution of mRS scores using binary logistic regression and ordinal logistic regression, respectively. There are two stratified methods for 2hPBG values in the present study. Four models were conducted to estimate the associations between the 2hPBG and the outcome individually in each stratified methods. In Model 1, no statistical correction has been made. Model 2 included age and sex, which cannot be reversed factors. In Model 3, confounder variables identified as significant in the univariate analyses (*P* < 0.05) were entered into logistic regression analyses. Model 4 included all of the potential factors.

The software package SPSS 17.0 was used to perform statistical analyses.

## Results

### Baseline Demographics and Clinical Characteristics According to 2hPBG Quartiles

**Figure 2:**
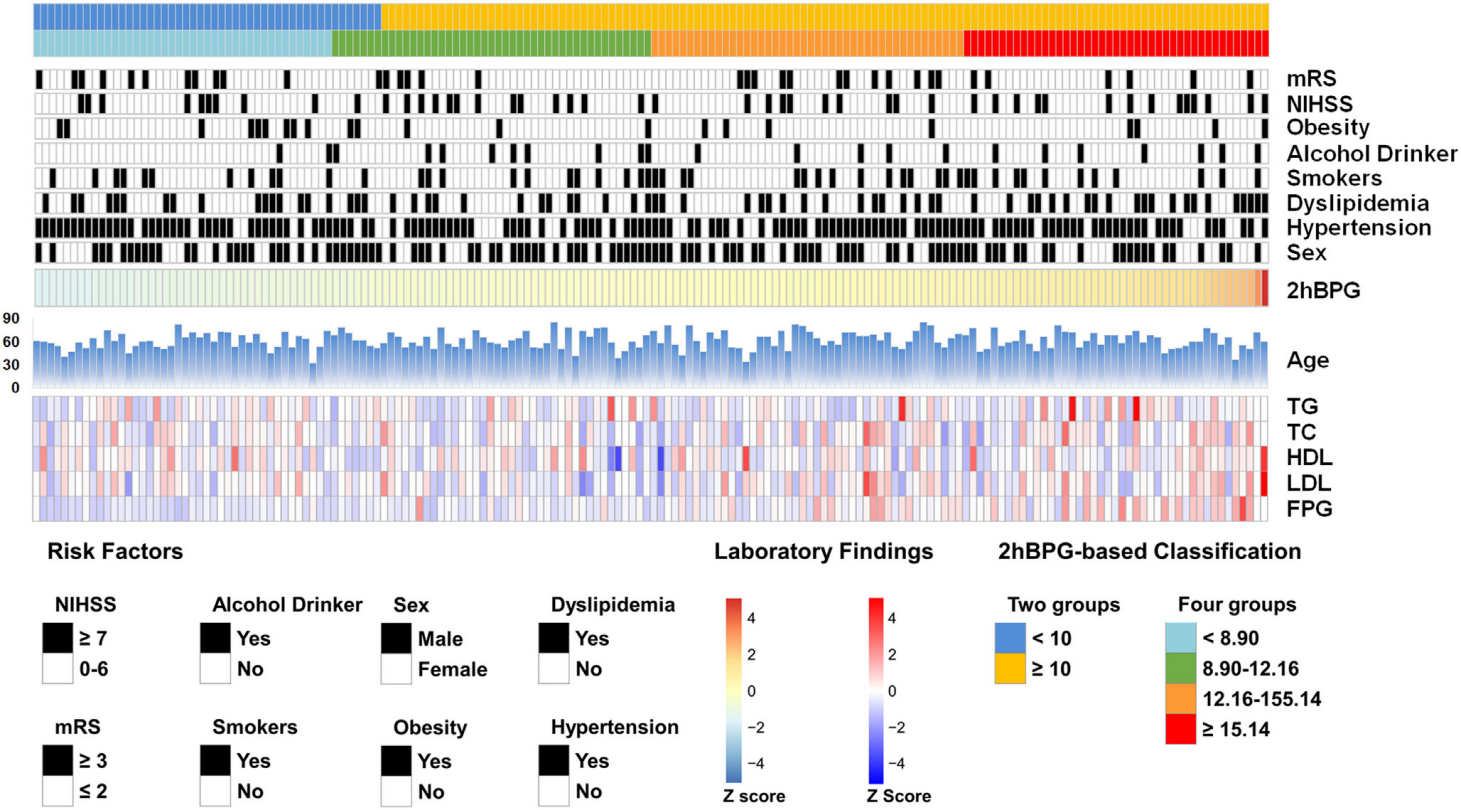
The Baseline demographics and clinical characteristics of enrolled patients. The risk factors and laboratory findings were normalized (*Z*-score) and presented according to 2hBPG level. 2hPBG, 2-h postprandial blood glucose; TG, triglyceride; TC, total cholesterol; HDL-C, high-density lipoprotein cholesterol; LDL-C, low-density lipoprotein cholesterol; FPG, fasting plasma glucose; NIHSS, National Institute of Health stroke scale; mRS, modified Rankin scale.

Table 1 and Figure 2 demonstrate the clinical characteristics of patients according to 2hPBG levels. A total of 174 patients [107 men (61.5%); mean age, 62.64 ± 10.92 years; range, 33–86 years] were included in the study. The 2hPBG median and inter-quartile spacing of all patients were 12.16 mmol/L (8.90, 15.14) (range, 4.44–39.40 mmol/L). The 2hPBG levels were <10 mmol/L in 49 patients (28.2%) and ≥10 mmol/L in 125 patients (71.8%). FBG levels tended to increase with higher 2hPBG levels. The percentages of stroke severity were equally distributed among the 2hPBG quartiles. There was no statistically significant difference among the stratifications with regard to antiplatelet therapy.

**Table 1:** Baseline demographics and clinical characteristics according to 2hPBG.

	2hPBG (mmol/L)							
	<8.90 (*n* = 42)	≥8.90 (*n* = 45)	≥12.16 (*n* = 44)	≥15.14 (*n* = 43)	*P*Value	<10.00 (*n* = 49)	≥10.00 (*n* = 125)	*P*-Value
Age, years	62.93 ± 10.07	63.53 ± 10.81	63.16 ± 11.36	60.91 ± 11.56	0.683	63.39 ± 9.77	62.35 ± 11.36	0.575
Male sex, *n* (%)	24 (57.1)	32 (71.1)	29 (65.9)	22 (51.2)	0.222	31 (63.3)	76 (60.8)	0.764
Risk factors								
Hypertension, *n* (%)	36 (85.7)	31 (68.9)	35 (79.5)	33 (76.7)	0.300	41 (83.7)	94 (75.2)	0.228
Dyslipidemia, *n* (%)	16 (38.1)	16 (35.6)	16 (36.4)	21 (48.8)	0.557	19 (38.8)	50 (40.0)	0.882
Smokers, *n* (%)	11 (26.2)	12 (26.7)	15 (34.1)	12 (27.9)	0.836	12 (24.5)	38 (30.4)	0.438
Alcohol drinkers, *n* (%)	2 (4.8)	9 (20.0)	5 (11.4)	7 (16.3)	0.179	3 (6.1)	20 (16.0)	0.084
Obesity, *n* (%)	9 (21.4)	5 (11.1)	4 (9.1)	4 (9.3)	0.265	11 (22.4)	11 (8.8)	0.015
Laboratory findings								
TG, mmol/L	1.84 ± 0.73	1.86 ± 0.91	1.82 ± 0.92	2.15 ± 1.28	0.376	1.85 ± 0.73	1.94 ± 1.06	0.588
TC, mmol/L	4.96 ± 0.90	4.72 ± 0.96	5.03 ± 1.23	5.35 ± 1.60	0.136	4.92 ± 0.89	5.06 ± 1.32	0.423
HDL-C, mmol/L	1.04 ± 0.25	0.98 ± 0.27	0.99 ± 0.31	1.04 ± 0.29	0.649	1.00 ± 0.26	1.01 ± 0.29	0.803
LDL-C, mmol/L	3.05 ± 0.78	2.76 ± 0.86	3.16 ± 1.12	3.30 ± 1.22	0.107	3.03 ± 0.75	3.09 ± 1.11	0.709
FPG, mmol/L	6.27 ± 1.12	7.07 ± 1.81	8.53 ± 2.29	10.60 ± 4.83	<0.001	6.30 ± 1.08	8.82 ± 3.57	<0.001
2hPBG, mmol/L	6.66 ± 1.27	10.82 ± 0.85	13.63 ± 0.90	19.68 ± 4.45	<0.001	7.04 ± 1.52	14.94 ± 4.48	<0.001
NIHSS, *n* (%)					0.546			0.219
0–6	33 (78.6)	31 (68.9)	34 (77.3)	29 (67.4)		39 (79.6)	88 (70.4)	
≥7	9 (21.4)	14 (31.1)	10 (22.7)	14 (32.6)		10 (20.4)	37 (29.6)	

*2hPBG, 2-h postprandial blood glucose; TG, triglyceride; TC, total cholesterol; HDL-C, high-density lipoprotein cholesterol; LDL-C, low-density lipoprotein cholesterol; FPG, fasting plasma glucose; NIHSS, National Institute of Health stroke scale*.

### Association between 2hPBG Levels and Functional Outcomes

Table 2 compares characteristics between the favorable outcome and unfavorable outcome groups. Among all patients, there were 139 patients (79.9%) with favorable outcomes and 35 patients (20.1%) with poor outcomes. The percentages of patients with higher NIHSS scores were significantly different according to mRS scores (*P* < 0.001). In addition, patients with unfavorable outcomes were less likely to be smokers and alcohol drinkers.

**Table 2:** Comparison of the risk factors between different groups classified by outcomes.

	mRS ≤ 2 (*n* = 139)	mRS ≥ 3 (*n* = 35)	*P*-Value
Age, years (median values)	61.94 ± 10.80	65.43 ± 11.11	0.091
Male sex, *n* (%)	85 (61.2)	22 (62.9)	0.853
Hypertension, *n* (%)	109 (78.4)	26 (74.3)	0.600
Dyslipidemia, *n* (%)	57 (41.0)	12 (34.3)	0.468
Smokers, *n* (%)	45 (32.4)	5 (14.3)	0.035
Alcohol drinkers, *n* (%)	22 (15.8)	1 (2.9)	0.043
Obesity, *n* (%)	18 (12.9)	4 (11.4)	0.809
FPG, mmol/L (median values)	8.18 ± 3.46	7.84 ± 2.44	0.578
2hPBG, *n* (%)			0.197
<8.90	31 (22.3)	11 (31.4)	
≥8.90	39 (28.1)	6 (17.1)	
≥12.16	32 (23.0)	12 (34.3)	
≥15.14	37 (26.6)	6 (17.1)	
2hPBG, *n* (%)			0.367
<10.00	37 (26.6)	12 (34.3)	
≥10.00	102 (73.4)	23 (65.7)	
NIHSS, *n* (%)			<0.001
0–6	111 (79.9)	16 (45.7)	
≥7	28 (20.1)	19 (54.3)	

*mRS, modified Rankin scale; FPG, fasting plasma glucose; 2hPBG, 2-h postprandial blood glucose; NIHSS, National Institute of Health stroke scale*.

**Figure 3:**
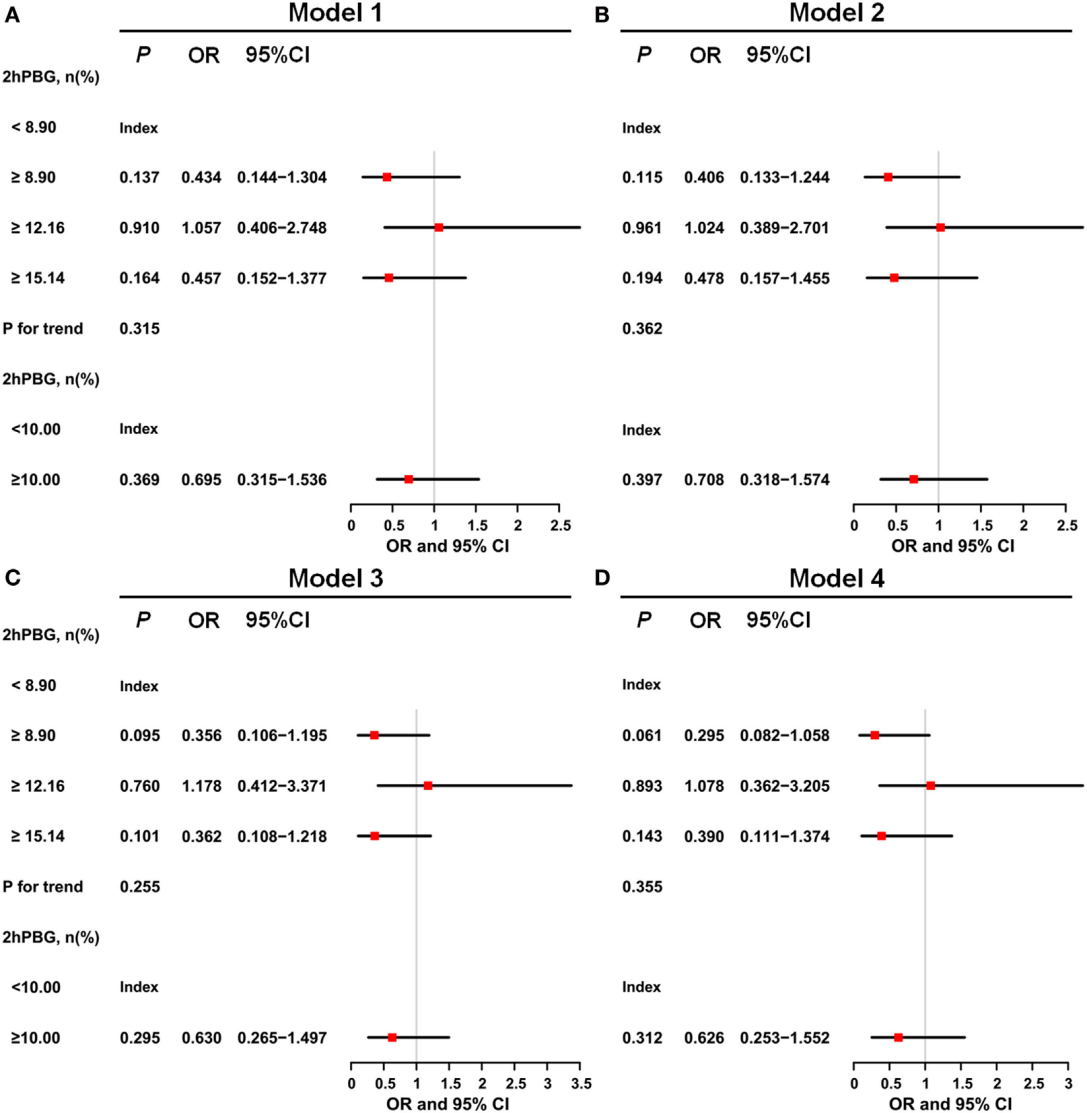
Odds ratios for 2-h postprandial blood glucose (2hPBG)-based model. **(A)** For Model 1, the results indicated that compared with the lowest level of 2hPBG (<8.90 mmol/L), 2hPBG in other three higher quartiles (8.90 to <12.16, 12.16 to <15.14, and ≥15.14 mmol/L) had no significant trend in increasing risk of poor functional outcome. **(B)** Model 2 included age and sex. **(C)** Model 3 included the variables of confounders that were identified as significant in the univariate analyses (smoking, alcohol consumption, and National Institute of Health stroke scale score). **(D)** Model 4 included the same variables in Model 2, Model 3, and hypertension, dyslipidemia, and obesity.

Figure 3 shows the results of the binary logistic regression analyses of 2hPBG levels and 3-month functional outcomes. The results indicated that compared with the lowest level of 2hPBG (<8.90 mmol/L), 2hPBG in the three higher quartiles (8.90 to <12.16, 12.16 to <15.14, and ≥15.14 mmol/L) had no significant trend in increasing risk of poor functional outcome. No statistical correction was made Model 1. Model 2 included age and sex, and Model 3 included the variables of confounders that were identified as significant in univariate analysis (smoking, alcohol consumption, and NIHSS score). Model 4 included the same variables as Models 2 and 3. The *P* for trend with respect to hypertension, dyslipidemia, and obesity was 0.315, 0.362, 0.255, and 0.355, respectively, for the four groups. Because lowering postprandial blood glucose to <10 mmol/L was recommended in clinical practice, we further compared patients with 2hPBG levels <10 mmol/L to those with 2hPBG levels ≥10 mmol/L; the latter did not have a significantly higher risk of poor outcome after adjusting for confounders (*P* for trend of Model 1, Model 2, Model 3, and Model 4 was 0.369, 0.397, 0.295, and 0.312, respectively).

**Figure 4:**
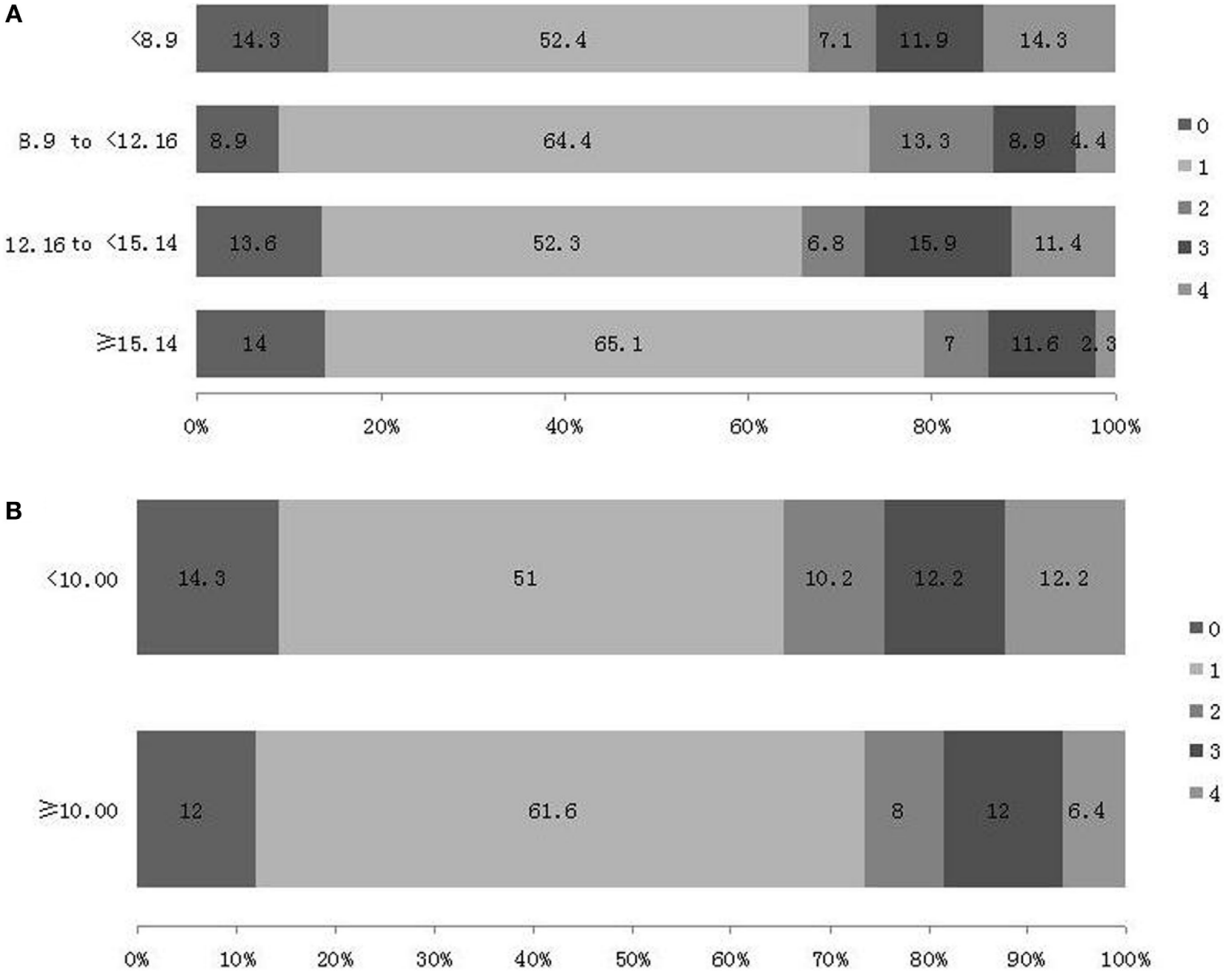
Distribution of modified Rankin Scale (mRS) score at 3 month after stroke [**(A)** based on 2-h postprandial blood glucose (2hPBG) values according to quartiles and **(B)** based on 2hPBG values according to clinical glycemic recommendations].

Similarly, the negative results appeared in the ordinal logistic regression (Figure 4). No significant association has be shown between 2hPBG and the distribution of mRS in Model 3 (8.90 to <12.16, OR 0.636, 95% CI, −1.310–0.405, *P* = 0.301; 12.16 to <15.14, OR 1.012, 95% CI, −0.834–0.857, *P* = 0.978; ≥15.14, OR 0.449, 95% CI, −1.670–0.070, *P* = 0.071), and in Model 4 (8.90 to <12.16, OR 0.530, 95% CI, −1.514–0.245, *P* = 0.157; 12.16 to <15.14, OR 0.868, 95% CI, −0.998–0.717, *P* = 0.748; ≥15.14, OR 0.416, 95% CI, −1.772–0.015, *P* = 0.054).

In current study, the stroke severity assessed by NIHSS scores shown the positive results both in binary and ordinal logistic regression. In Model 3, compared with the 0–6 stratification, the 7–14 stratification was associated with a risk of poor outcome (OR 5.561, 95% CI, 2.356–13.126, *P* < 0.001 in binary logistic regression; OR 6.896, 95% CI, 1.225–2.637, *P* < 0.001 in ordinal logistic regression). In Model 4, compared with the 0–6 stratification, the 7–14 stratification was also associated with the risk of poor outcome (OR 6.062, 95% CI, 2.478–14.832, *P* < 0.001 in binary logistic regression; OR 7.471, 95% CI, 1.291–2.732, *P* < 0.001 in ordinal logistic regression).

## Discussion

It has been established that pre-stroke glycemic control status, which is usually reflected by hemoglobin A1c (HbA1c), is a more stable way to evaluate glycemic control than random blood glucose testing (12). It has been shown that HbA1c is also an independent prognostic factor of mortality and functional outcome among patients diagnosed with ischemic stroke (9, 10). In our previous study, we found that elevated HbA1c levels were associated with unfavorable functional outcomes 3 months after stroke onset among patients with SAO (10). Furthermore, hyperglycemia at admission, measured using FBG or random blood glucose levels, was associated with poor outcomes in acute stroke patients. Hyperglycemia at admission is common, with up to 68% of stroke patients presenting with hyperglycemia during the acute stroke phase (20); a similar result was found in our study, in which 65.8% of patients had diabetes or impaired glucose regulation. The mechanisms underlying the association between hyperglycemia at admission and poor outcome involve many factors, such as direct neuronal toxicity *via* the induction of oxidative stress and inflammation (21, 22) and the expansion of the penumbra area through procoagulant effects, which interferes with blood supply (23–25).

However, the effects of hyperglycemia at admission might differ according to TOAST stroke subtype, as hyperglycemia was associated with poor outcomes among patients with large-artery atherosclerosis (8, 11, 26) but not among those with SAO (11–13). The finding that hyperglycemia has protective effects against stroke was also confirmed in animal end-artery infarct models (27–29), and these end-artery infarct models mimic the SAO stroke subtype in humans. The reason why hyperglycemia at admission is associated with different effects on different stroke subtypes is not clear, partially because of the existence of different inflammation characteristics among TOAST subtypes (30, 31).

Recently, the role of 2hPBG levels in the outcomes of SAO patients has received more attention, because 2hPBG levels are an independent risk factor for vascular diseases, while FBG levels are not (15, 16, 32). The DECODE Study found that, compared with FBG levels, 2hPBG levels were a superior predictive factor for all-cause mortality (HR 1.73 [1.45–2.06]) and cardiovascular disease (HR 1.40 [1.02–1.92]) (33). Levels of 2hPBG may be a better indicator of glucose control than FBG and random blood glucose levels because it may not be affected by some factors, such as reinstitution of oral feeding or intravenous administration of glucose-containing fluids. However, physicians continue to rely on FBG and HbA1c levels as indicators for disease management.

In the present study, we found that patients with higher 2hPBG levels (8.90 to <12.16, 12.16 to <15.14, and ≥15.14 mmol/L) had no significant trend for lower risk of a favorable outcome compared with patients with the lowest 2hPBG levels, and compared with patients with 2hPBG levels <10 mmol/L, those with 2hPBG levels ≥10 mmol/L did not have a significant risk of poor outcome after adjusting for confounders. Our findings were in line with those reported in the study by Bruno et al. (11); the only difference between the two studies was the latter study included stroke patients both with and without diabetes. The reason why higher 2hPBG levels have no effects on functional outcome among SAO patients with diabetes is still under investigation. One possible explanation for this link is that SAO patients with diabetes are more prone to develop a pro-inflammatory, hypercoagulable state compared with patients without diabetes. As a result, patients with diabetes may develop a tolerance to fluctuating blood glucose levels (34, 35).

Our finding has important clinical implications. It has been shown that acarbose decreased the incidence of major adverse cardiovascular events and slowed the progression of carotid intima-middle thickness through lowering postprandial blood glucose (36, 37). However, based on the findings of present study, it should be noted that lowering postprandial blood glucose to some extent in the acute phase, regardless of stroke subtype, may adversely affect the outcome, especially in SAO patients (27–29). For patients with SAO, it is important to routinely measure 2hPBG levels and to avoid excessive reduction of PBG levels, as this may benefit early intervention in clinical practice.

The present study had the following limitations. First, as all the subjects were recruited from a single hospital in north China, the sample size is too small to determine clinical relevance. Second, blood glucose levels were based on a single measurement; therefore, there may be an inevitable selection bias. Third, we could not test 2hPBG levels during the follow-up period. Future studies with larger patient populations are necessary to evaluate the effect of diabetes and 2hPBG levels after SAO.

## Conclusion

In this study, higher 2hPBG levels were not associated with unfavorable functional outcomes 3 months after stroke onset in SAO patients with diabetes.

## Ethics Statement

This study was approved by the ethics committee of Tianjin Huanhu Hospital. Written informed consent was obtained from the individuals and their families at the beginning of the study.

## Author Contributions

JW contributed to the conception and design of the work and contributed in revising the work for important intellectual content. JL and DH contributed in the data acquisition. JL, DH, and YG contributed in the analysis and interpretation of data for the work. JL and DH contributed in drafting the work. All authors approved the final version to be published, and agreed to be accountable for all aspects of the work in ensuring that questions related to the accuracy or integrity of any part of the work are appropriately investigated and resolved.

## Conflict of Interest Statement

The authors declare that the research was conducted in the absence of any commercial or financial relationships that could be construed as a potential conflict of interest.

## References

[1] Adams HP Jr Bendixen BH Kappelle LJ Biller J Love BB Gordon DL Classification of subtype of acute ischemic stroke. Definitions for use in a multicenter clinical trial. TOAST. Trial of Org 10172 in Acute Stroke Treatment. Stroke (1993) 24:35–41. 10.1161/01.STR.24.1.35 7678184

[2] Yu C An Z Zhao W Wang W Gao C Liu S Sex differences in stroke subtypes, severity, risk factors, and outcomes among elderly patients with acute ischemic stroke. Front Aging Neurosci (2015) 7:174. 10.3389/fnagi.2015.00174 26441636 PMC4561826

[3] Lv P Jin H Liu Y Cui W Peng Q Liu R Comparison of risk factor between Lacunar stroke and large artery atherosclerosis stroke: a cross-sectional study in China. PLoS One (2016) 11:e0149605. 10.1371/journal.pone.0149605 26934734 PMC4774914

[4] Jia Q Liu G Zheng H Zhao X Wang C Wang Y Impaired glucose regulation predicted 1-year mortality of Chinese patients with ischemic stroke: data from abnormal glucose regulation in patients with acute stroke across China. Stroke (2014) 45:1498–500. 10.1161/STROKEAHA.113.002977 24676777

[5] Hu GC Hsieh SF Chen YM Hsu HH Hu YN Chien KL. Relationship of initial glucose level and all-cause death in patients with ischaemic stroke: the roles of diabetes mellitus and glycated hemoglobin level. Eur J Neurol (2012) 19:884–91. 10.1111/j.1468-1331.2011.03647.x 22289016

[6] Paciaroni M Agnelli G Caso V Corea F Ageno W Alberti A Acute hyperglycemia and early hemorrhagic transformation in ischemic stroke. Cerebrovasc Dis (2009) 28:119–23. 10.1159/000223436 19506370

[7] Vancheri F Curcio M Burgio A Salvaggio S Gruttadauria G Lunetta MC Impaired glucose metabolism in patients with acute stroke and no previous diagnosis of diabetes mellitus. QJM (2005) 98:871–8. 10.1093/qjmed/hci134 16239309

[8] Frank S Gonzalez K Lee-Ang L Young MC Tamez M Mattei J. Diet and sleep physiology: public health and clinical implications. Front Neurol (2017) 8:393. 10.3389/fneur.2017.00393 28848491 PMC5554513

[9] Wu S Wang C Jia Q Liu G Hoff K Wang X HbA1c is associated with increased all-cause mortality in the first year after acute ischemic stroke. Neurol Res (2014) 36:444–52. 10.1179/1743132814Y.0000000355 24649851

[10] Gao Y Jiang L Wang H Yu C Wang W Liu S Association between elevated hemoglobin A1c levels and the outcomes of patients with small-artery occlusion: a hospital-based study. PLoS One (2016) 11:e0160223. 10.1371/journal.pone.0160223 27486868 PMC4972422

[11] Bruno A Biller J Adams HP Jr Clarke WR Woolson RF Williams LS Acute blood glucose level and outcome from ischemic stroke. Trial of ORG 10172 in Acute Stroke Treatment (TOAST) Investigators. Neurology (1999) 52:280–4. 10.1212/WNL.52.2.280 9932944

[12] Uyttenboogaart M Koch MW Stewart RE Vroomen PC Luijckx GJ De Keyser J Moderate hyperglycaemia is associated with favourable outcome in acute lacunar stroke. Brain (2007) 130:1626–30. 10.1093/brain/awm087 17525141

[13] Fang Y Zhang S Wu B Liu M. Hyperglycaemia in acute lacunar stroke: a Chinese hospital-based study. Diab Vasc Dis Res (2013) 10:216–21. 10.1177/1479164112459663 23079541

[14] Jia Q Zheng H Zhao X Wang C Liu G Wang Y Abnormal glucose regulation in patients with acute stroke across China: prevalence and baseline patient characteristics. Stroke (2012) 43:650–7. 10.1161/STROKEAHA.111.633784 22267822

[15] Hanefeld M Koehler C Henkel E Fuecker K Schaper F Temelkova-Kurktschiev T. Post-challenge hyperglycaemia relates more strongly than fasting hyperglycaemia with carotid intima-media thickness: the RIAD Study. Risk factors in impaired glucose tolerance for atherosclerosis and diabetes. Diabet Med (2000) 17:835–40. 10.1046/j.1464-5491.2000.00408.x 11168325

[16] American Diabetes Association. Standards of medical care in diabetes – 2013. Diabetes Care (2013) 36(Suppl 1):S11–66. 10.2337/dc13-S011 23264422 PMC3537269

[17] Hong Y Yang X Zhao W Zhang X Zhao J Yang Y Sex differences in outcomes among stroke survivors with non-valvular atrial fibrillation in China. Front Neurol (2017) 8:166. 10.3389/fneur.2017.00166 28496431 PMC5406396

[18] Jiang B Sun H Ru X Sun D Chen Z Liu H Prevalence, incidence, prognosis, early stroke risk, and stroke-related prognostic factors of definite or probable transient ischemic attacks in China. Front Neurol (2017) 8:309 10.3389/fneur.2017.00309 28713329 PMC5491639

[19] Jia Q Liu G Zheng H Zhao X Wang C Wang Y Investigators for the survey on abnormal glucose regulation in patients with acute stroke across China. Impaired glucose regulation predicted 1-year mortality of Chinese patients with ischemic stroke: data from abnormal glucose regulation in patients with acute stroke across China. Stroke (2014) 45:1498–500. 10.1161/STROKEAHA.113.002977 24676777

[20] MacDougall NJ Muir KW Hyperglycaemia and infarct size in animal models of middle cerebral artery occlusion: systematic review and meta-analysis. J Cereb Blood Flow Metab (2011r) 31:807–18. 10.1038/jcbfm.2010.210 21157471 PMC3063635

[21] Baird TA Parsons MW Barber PA Butcher KS Desmond PM Tress BM The influence of diabetes mellitus and hyperglycaemia on stroke incidence and outcome. J Clin Neurosci (2002) 9:618–26. 10.1054/jocn.2002.1081 12604269

[22] Wu S Ren J. Benfotiamine alleviates diabetes-induced cerebral oxidative damage independent of advanced glycation end-product, tissue factor and TNF-alpha. Neurosci Lett (2006) 394:158–62. 10.1016/j.neulet.2005.10.022 16260089

[23] Garg R Chaudhuri A Munschauer F Dandona P Hyperglycemia, insulin, and acute ischemic stroke: a mechanistic justification for a trial of insulin infusion therapy. Stroke (2005) 37:267–73. 10.1161/01.STR.0000195175.29487.30 16306459

[24] Shimoyama T Shibazaki K Kimura K Uemura J Shiromoto T Watanabe M Admission hyperglycemia causes infarct volume expansion in patients with ICA or MCA occlusion: association of collateral grade on conventional angiography. Eur J Neurol (2013) 20:109–16. 10.1111/j.1468-1331.2012.03801.x 22747888

[25] Rosso C Pires C Corvol JC Baronnet F Crozier S Leger A Hyperglycaemia, insulin therapy and critical penumbral regions for prognosis in acute stroke: further insights from the INSULINFARCT trial. PLoS One (2015) 10:e0120230. 10.1371/journal.pone.0120230 25793765 PMC4368038

[26] de Courten-Myers G Myers RE Schoolfield L. Hyperglycemia enlarges infarct size in cerebrovascular occlusion in cats. Stroke (1988) 19:623–30. 10.1161/01.STR.19.5.623 3363596

[27] Kraft SA Larson CP Jr Shuer LM Steinberg GK Benson GV Pearl RG. Effect of hyperglycemia on neuronal changes in a rabbit model of focal cerebral ischemia. Stroke (1990) 21:447–50. 10.1161/01.STR.21.3.447 2309269

[28] Prado R Ginsberg MD Dietrich WD Watson BD Busto R. Hyperglycemia increases infarct size in collaterally perfused but not end-arterial vascular territories. J Cereb Blood Flow Metab (1988) 8:186–92. 10.1038/jcbfm.1988.48 3343293

[29] Ginsberg MD Prado R Dietrich WD Busto R Watson BD. Hyperglycemia reduces the extent of cerebral infarction in rats. Stroke (1987) 18:570–4. 10.1161/01.STR.18.3.570 3109078

[30] Qiu R Gao Y Hou D Wang Y Yu C Wang W Association between hs-CRP Levels and the Outcomes of Patients with Small-Artery Occlusion. Front Aging Neurosci (2016) 8:191 10.3389/fnagi.2016.00191 27555819 PMC4977298

[31] Zeng L He X Liu J Wang L Weng S Wang Y Differences of circulating inflammatory markers between large- and small vessel disease in patients with acute ischemic stroke. Int J Med Sci (2013) 10:1399–405. 10.7150/ijms.6652 23983602 PMC3753418

[32] Ceriello A Davidson J Hanefeld M Leiter L Monnier L Owens D Postprandial hyperglycaemia and cardiovascular complications of diabetes: an update. Nutr Metab Cardiovasc Dis (2006) 16:453–6. 10.1016/j.numecd.2006.05.006 16934443

[33] DECODE Study Group, The European Diabetes Epidemiology Group. Glucose tolerance and cardiovascular mortality: comparison of fasting and 2-hour diagnostic criteria. Arch Intern Med (2001) 161:397–405. 10.1001/archinte.161.3.397 11176766

[34] Saito M Ishimitsu T Minami J Ono H Ohrui M Matsuoka H. Relations of plasma high-sensitivity C-reactive protein to traditional cardiovascular risk factors. Atherosclerosis (2003) 167:73–9. 10.1016/S0021-9150(02)00380-5 12618270

[35] Ormstad H Aass HC Lund-Sørensen N Amthor KF Sandvik L. Serum levels of cytokines and C-reactive protein in acute ischemic stroke patients, and their relationship to stroke lateralization, type, and infarct volume. J Neurol (2011) 258(4):677–85. 10.1007/s00415-011-6006-0 21424610 PMC3065641

[36] Hanefeld M Chiasson JL Koehler C Henkel E Schaper F Temelkova-Kurktschiev T. Acarbose slows progression of intima-media thickness of the carotid arteries in subjects with impaired glucose tolerance. Stroke (2004) 35:1073–8. 10.1161/01.STR.0000125864.01546.f2 15073402

[37] Yun P Du AM Chen XJ Liu JC Xiao H. Effect of acarbose on long-term prognosis in acute coronary syndromes patients with newly diagnosed impaired glucose tolerance. J Diabetes Res (2016) 2016:1602083. 10.1155/2016/1602083 26770983 PMC4684859

